# Ultrasensitive Measurement of Ca^2+^ Influx into Lipid Vesicles Induced by Protein Aggregates

**DOI:** 10.1002/anie.201700966

**Published:** 2017-05-05

**Authors:** Patrick Flagmeier, Suman De, David C. Wirthensohn, Steven F. Lee, Cécile Vincke, Serge Muyldermans, Tuomas P. J. Knowles, Sonia Gandhi, Christopher M. Dobson, David Klenerman

**Affiliations:** ^1^ Department of Chemistry University of Cambridge Lensfield Road Cambridge CB2 1EW UK; ^2^ Laboratory of Cellular and Molecular Immunology Vrije Universiteit Brussel Brussels Belgium; ^3^ Department of Molecular Neuroscience Institute of Neurology University College London Queen Square London WC1N 3BG UK

**Keywords:** Alzheimer's disease, fluorescence imaging, nano-scale biophysics, neurodegeneration, protein aggregation

## Abstract

To quantify and characterize the potentially toxic protein aggregates associated with neurodegenerative diseases, a high‐throughput assay based on measuring the extent of aggregate‐induced Ca^2+^ entry into individual lipid vesicles has been developed. This approach was implemented by tethering vesicles containing a Ca^2+^ sensitive fluorescent dye to a passivated surface and measuring changes in the fluorescence as a result of membrane disruption using total internal reflection microscopy. Picomolar concentrations of Aβ42 oligomers could be observed to induce Ca^2+^ influx, which could be inhibited by the addition of a naturally occurring chaperone and a nanobody designed to bind to the Aβ peptide. We show that the assay can be used to study aggregates from other proteins, such as α‐synuclein, and to probe the effects of complex biofluids, such as cerebrospinal fluid, and thus has wide applicability.

The conversion of soluble proteins into fibrillar amyloid deposits is a hallmark of a range of increasingly prevalent neurodegenerative disorders including Alzheimer's Disease (AD) and Parkinson Disease (PD).^1^, ^2^, ^3^, ^4^ It has been reported that the species formed during the aggregation reactions have a toxic effect on cells.^5^, ^6^, ^7^, ^8^ Several possible mechanisms of cellular damage have been identified with relative contributions that depend on both the concentrations and the types of aggregates present.^1^, ^2^, ^3^, ^4^, ^5^, ^6^, ^7^, ^8^, ^9^, ^10^, ^11^ The aggregation process that results in the formation of oligomers may contribute to cellular damage.^12^, ^13^, ^14^, ^15^ Some of these mechanisms are thought to involve specific binding to receptors on the cell membrane^1^, ^5^, ^9^ while others appear to be the consequence of non‐specific membrane disruption.^1^, ^3^, ^5^, ^9^, ^16^ A body of data suggests that small soluble aggregates, often called oligomers, rather than mature fibrils, are able to cause the membrane to become permeable to Ca^2+^ resulting in Ca^2+^ influx and the disruption of Ca^2+^ homeostasis.^2^, ^3^, ^5^, ^9^, ^17^, ^18^ Therefore, it is important to quantify and characterize these species within aggregation mixtures in vitro as well as to be able to perform measurements in biological samples, such as cerebrospinal fluid (CSF).^19^ However, these oligomers are known to be present at low abundance and to be highly heterogeneous in nature, in terms of both their size and structure.^1^, ^2^, ^3^, ^4^, ^9^, ^10^, ^16^

Herein we describe the development and application of a quantitative method based on the extent of disruption of membranes by protein aggregates monitored by measurement of the resulting Ca^2+^ entry into vesicles. Methods to detect membrane disruption in bulk solution, based on measuring dye leakage, are valuable for high μm concentrations of toxic species,^20^ but we utilize nanosized vesicles filled with a dye whose fluorescence increases on binding Ca^2+^ ions. The entry of a small number of Ca^2+^ ions into such small volumes leads to a significant change in concentration, enabling high resolution measurements to be made. For example, if a single Ca^2+^ ion enters an individual vesicle with a diameter of 200 nm, the concentration of Ca^2+^ will change by approximately 400 nm, which can readily be detected using highly sensitive fluorescence microscopy techniques. We designed our assay to achieve an optimal combination of sensitivity and dynamic range by preparing vesicles of an appropriate diameter encapsulated with the optimal concentrations of the Ca^2+^ binding dye, Cal‐520 (see Supporting Information Note 1 and 2 and Supporting Information Figure S1). We immobilized thousands of single vesicles with a diameter of 200 nm containing dye molecules on the glass coverslides and imaged the Ca^2+^ influx into hundreds of individual vesicles per field of view using total internal reflection fluorescence microscopy (TIRFM; Figure 1, Figure S2). This method enables the detection of small changes in the Ca^2+^ concentration on many vesicles in parallel leading to a high degree of accuracy.

**Figure 1:**
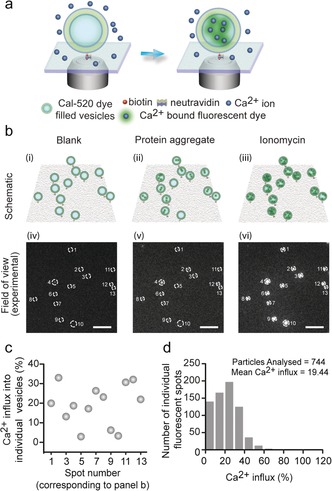
Quantitative high‐throughput fluorescence imaging of Ca^2+^ ion influx into individual surface‐tethered vesicles imaged using TIRF microscopy. a) Individual vesicles filled with the fluorescent dye Cal‐520 are immobilized on a polymer‐passivated (PLL‐g‐PEG/PLL‐g‐PEG‐biotin) glass cover slide through biotin‐neutravidin tethering. Addition of membrane‐disrupting species (e.g. protein aggregates) leads to Ca^2+^ influx into vesicles resulting in an increase in the localized fluorescence intensity. b) Identical positions of the coverslides are imaged under three different conditions, shown schematically (i)–(iii) and as TIRF images (iv)–(vi). Images are acquired in the presence of only Ca^2+^ buffer [(i) and (iv)], followed by the addition of protein aggregates [(ii) and (v)], and then the addition of the ionophore ionomycin [(iii) and (vi)]. The TIRF images were averaged over 50 frames with an exposure time of 50 ms each without further image processing. Individual vesicles containing Cal‐520 dye molecules appear as localized bright spots under 488‐nm illumination. In the presence of Ca^2+^ buffer alone, the intensity of a vesicle is comparable to that of the background due to no or minimal Ca^2+^ influx into the vesicles. Addition and incubation with protein aggregates causes a significant increase in the fluorescence of some of the vesicles and subsequent addition of ionomycin results in saturation of all vesicles by Ca^2+^, causing detection of a maximum value of the fluorescence signal. All the images are shown with equal contrast. The scale bar: 3 μm. c) Ca^2+^ influx into 13 individual vesicles as shown in (b) (iv)–(vi). The percentage of Ca^2+^ influx in each vesicle was calculated using: (*F*_aggregate_−*F*_blank_)*100/ (*F*_ionomycin_−*F*_aggregate_), where *F*_blank_, *F*_aggregate_, *F*_ionomycin_ represent the fluorescence in the presence of Ca^2+^ containing buffer, a solution containing protein samples (e.g. aggregates), and ionomycin, respectively. d) Histogram showing the distribution of the percentage of Ca^2+^ influx into 744 individual vesicles after the addition of aliquots taken from an aggregation reaction of Aβ42 with an average Ca^2+^ influx of 19.44 %.

The vesicles used were composed of 1‐palmitoyl‐2‐oleoyl‐sn‐glycero‐3‐phosphocholine (POPC) and biotinylated POPC (at a ratio of 100:1) filled with the Ca^2+^‐sensitive Cal‐520 dye and prepared by extrusion.^21^ To remove non‐encapsulated dye molecules from the solution, size exclusion chromatography was carried out (see Supporting Information, Figure S3) prior to tethering the vesicles onto PEG (PLL‐g‐PEG: PLL‐g‐PEG‐biotin 100:1) coated glass coverslides via biotin‐neutravidin linkages;^22^, ^23^, ^24^ such a process has been reported to maintain ion permeability,^22^ spherical morphology,^23^ and membrane diffusivity^22^. The conditions were chosen to generate a low coverage of vesicles on the surface (ca. 1 %), enabling single vesicles to be monitored individually and analyzed.

We incubated these immobilized vesicles in Leibovitz's L‐15 medium containing Ca^2+^ at a concentration of 1.26 mm and imaged them using TIRFM (Figure 1 (i) and (iv), *F*_blank_). Typically, we imaged 16 fields of view (on a 4×4 grid) for each coverslide using a computer‐controlled automatic microscope stage, allowing the measurement of identical areas of the coverslide (Figure S4). We then added a solution containing the species of interest (e.g. protein aggregates) and re‐imaged the identical fields of view. If Ca^2+^ entered any given vesicle, an increase in the localized fluorescence intensity could be detected (Figure 1 b (ii) and (v), *F*_aggregate_). The fluorescence signals, before and after the addition of aliquots of the solution, were stable with time (Figures S5–S7) and hence the changes in the localized fluorescence can be attributed to species that disrupt the vesicles. To quantify the Ca^2+^ influx into individual vesicles, we added the cation transporting ionophore, ionomycin (Figures S6,S8), which allows Ca^2+^ to enter the vesicles to saturation. The maximum fluorescence intensity of each vesicle was then measured (Figure 1 b (iii) and (vi), *F*_ionomycin_) and the percentage of Ca^2+^ influx was calculated. This approach corrected for the fact that the number of Cal‐520 dye molecules in a vesicle is not constant, owing to variations in the efficiency of dye encapsulation^25^ and in vesicle size. The fluorescence intensity change was quantitatively converted into a percentage of Ca^2+^ influx for individual vesicles and then averaged over all the vesicles imaged (Figure 1, see Supporting Information for details).

We first carried out a series of experiments with solutions of the recombinant Aβ42 peptide (Figure 2 a), under conditions where the aggregation reaction has been found to be highly reproducible.^26^ Monomeric Aβ42 was purified (t_1_), and its addition to the vesicles resulted in no detectable increase in fluorescence, indicating that the monomeric protein does not induce Ca^2+^ influx (Figure 2 b).

**Figure 2:**
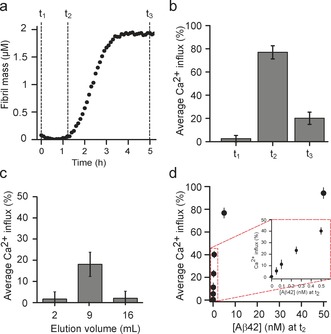
Ca^2+^ influx caused by aggregates of the Aβ42 peptide. a) Kinetic profile of Aβ42 aggregation under quiescent conditions at a concentration of 2 μm protein. The time points correspond to the start of the aggregation reaction (t_1_), the end of the lag‐phase (t_2_), and the plateau phase (t_3_), at each of which an aliquot was taken and diluted to a concentration of 5 nm (monomer equivalents). b) Ca^2+^ influx induced by an aggregation mixture at time points t_1_, t_2_, and t_3_; a Ca^2+^ influx of 100 % corresponds to the Ca^2+^ influx caused by ionomycin. c) Size exclusion chromatography of an aliquot taken at t_2_ and the degree of Ca^2+^ influx induced by different elution volumes was then tested. Oligomeric forms of the protein were found to elute at volumes of 7 to 12 mL and monomeric protein at a later elution volume of 14 to 17 mL. d) The dependence of Ca^2+^ influx on the total concentration of Aβ42 (in monomer equivalents) measured by serial dilution of an aliquot taken at t_2_. The error bars represent variations between different fields of view and the data correspond to averages over measurements of more than 700 vesicles. Inset: expansion of the low concentration region.

We then incubated 2 μm monomeric Aβ42 at 37 °C and took aliquots from the aggregation reaction corresponding to the end of the lag‐phase (t_2_). We detected an increase in the localized fluorescence due to the influx of Ca^2+^ resulting from the interaction of protein aggregates with the lipid bilayer. The aliquots taken from the aggregation reaction mixture had to be diluted 200 times to avoid saturation of individual vesicles with Ca^2+^, highlighting the sensitivity of our method. Note that oligomers of the Aβ peptide have been reported to be stable upon dilution^27^ and we observed their ability to induce Ca^2+^ influx into individual vesicles to remain constant for up to 2 h after dilution (Figure S9). We also tested aliquots corresponding to time points of an aggregation reaction at the plateau phase (t_3_), by which time most of the monomeric protein had been converted into fibrils.^14^ In this case, we detected a much lower fluorescence increase compared to that of aliquots of the sample taken at t_2_, although greater than that of aliquots at t_1_.

The contents of the aggregation solutions are heterogeneous, with the protein being present in monomeric and aggregated forms.^3^, ^4^ To determine the nature of the species of the protein causing Ca^2+^ influx we employed size exclusion chromatography to separate species of different size^14^ produced at the end of the lag‐phase (t_2_). We then probed the fluorescence changes arising from Ca^2+^ influx caused by the different fractions (Figure 2 c). We observed no detectable change in fluorescence with the fractions containing only monomeric protein (elution volume 16 mL), indicating that this species is not the component of the mixture causing Ca^2+^ ion influx. By contrast we observed a substantial level of fluorescence from the vesicles after the addition of those fractions that contain species of higher molecular weight than the monomeric protein, indicating that the observed Ca^2+^ influx is caused by Aβ42 oligomers.

To characterize the dynamic range and sensitivity of this assay we measured the dependence of the Ca^2+^ influx on the Aβ42 concentration, by carrying out a series of dilution experiments on a sample taken from an Aβ42 solution at a time point corresponding to t_2_. We found a linear dependence of the change in the fluorescence intensity caused by Aβ42 in the concentration range from 0 to 800 pm monomer equivalents, but noticed that the observed Ca^2+^ influx saturated at higher concentrations of Aβ42 (Figure 2 d). The method, therefore, enables us to measure quantitatively and reproducibly the Ca^2+^ influx induced by protein aggregates at picomolar concentrations (Figure S10,S11).

Next, we explored the potential of our method to test the effectiveness of two types of macromolecules, both of which have been attributed to bind to aggregated forms of Aβ42, in preventing the influx of Ca^2+^ ions. This approach has potential value for screening inhibitors in the search for effective therapeutic agents. We here studied the ability of the extracellular chaperone clusterin,^28^ and the nanobody Nb3,^29^ to reduce oligomer induced Ca^2+^ influx using aliquots taken from the Aβ42 aggregation reaction at time t_2_. We then diluted this solution containing pre‐formed oligomers to a concentration of 10 nm Aβ42 (monomer equivalents) and added 200 nm clusterin or 200 nm Nb3, respectively, both of which have been reported to bind to the Aβ peptide.^28^, ^29^ We incubated the samples for 15 min before assessing the ability of the excess concentrations of clusterin and Nb3 to counteract the membrane permeation caused by Aβ42 oligomers (Figure 3 a, see Supporting Information). We observed that both species reduced very significantly the degree of Ca^2+^ influx while incubation with an *anti*‐GFP antibody, as a control that does not bind to the Aβ peptide, had no detectable effect. These results are consistent with previous reports that clusterin^30^, ^31^ and Nb3 bind to oligomers of the Aβ peptide, resulting in the reduction of Ca^2+^ influx into neuronal cells.^7^, ^11^ We then carried out a series of dilution experiments to find the concentration required to reduce the Ca^2+^ influx by half; we obtained values of 0.1±0.02 nm and 18±3.4 nm for clusterin and Nb3, respectively (Figure 3 b,c). To our knowledge there is no reported value of the binding affinity of clusterin for Aβ42, but the off‐rate when bound to aggregates has been found to be very slow^27^ consistent with the tight binding observed in our experiments. The dissociation constant for Nb3 binding to monomeric Aβ42 has been reported to be 13 nm,^29^ a value comparable to that measured in the present study, suggesting there is no significant reduction in the accessibility of the epitope in the oligomeric species.

**Figure 3:**
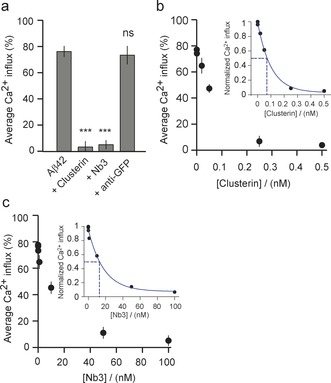
Testing the inhibition of aggregate‐induced Ca^2+^ influx resulting from the treatment of mixtures with binding proteins of aliquots of an Aβ42 aggregation reaction corresponding to time t_2_. a) Inhibition of Ca^2+^ influx resulting from Aβ42 aggregates by the chaperone clusterin (at 100 nm), the nanobody Nb3 (at 100 nm), and an *anti*‐GFP antibody (P<0.05 between Aβ42 and Aβ42 + antiGFP antibody). b) Concentration dependence of the inhibition by clusterin and c) by Nb3. Insets in (b) and (c) show the normalized Ca^2+^ influx with respect to the Ca^2+^ influx in the absence of clusterin and Nb3, respectively. Lines are guides to the eye. The error bars represent variations between different fields of view and the data correspond in each case to averages over measurements of more than 700 vesicles.

In an additional series of experiments, we have shown that the methodology can also be applied to the study of other proteins whose aggregation is associated with neurodegenerative conditions. We incubated α‐synuclein, the 140 residue natively unstructured protein whose aggregation is associated with PD, under conditions where it has previously been found to form oligomeric species, and observed a change in the fluorescence intensity (Figure S12) of individual vesicles similar to that described above for Aβ42.^18^, ^32^

We also examined the degree of Ca^2+^ influx induced by recombinant Aβ42 aggregates in the presence of a complex biological environment, such as CSF, which contains a multitude of different proteins and other macromolecules.^33^, ^34^ We took aliquots of an Aβ42 aggregation reaction at the time point corresponding to t_2_ and added them at varying concentrations to the human CSF of one individual (Figure S13). We observed an increase in the measured Ca^2+^ influx with Aβ42 concentrations of 100 pm and above. These results demonstrate that our method is sufficiently sensitive to measure the Ca^2+^ influx caused by protein aggregates at low concentrations in the presence of a complex human biofluid. Only small volumes of CSF were used in each experiment, 15 μL, allowing multiple measurements on a typical sample of CSF with a volume of 1 mL.

In conclusion, we have demonstrated that our single‐vesicle assay identifies oligomers as the form of protein aggregates in the complex aggregation mixtures of Aβ42 that cause Ca^2+^ influx. It also allows the extent of Ca^2+^ influx caused by aggregates in a sample to be quantified, which serves as a measure of the ability of aggregates to disrupt membrane integrity. The method also enables the effectiveness of reagents to reduce aggregate‐induced Ca^2+^ influx to be measured and can be applied directly to other proteins, such as α‐synuclein, and also to complex biofluids. The misfolding and aggregation of proteins has been associated with approximately fifty misfolding diseases^35^ for which our assay might be directly employed. Thus, application of the single‐vesicle assay to the field of protein misfolding diseases may greatly help global efforts to identify the pathogenic forms of proteins and to assist in the development of therapeutic agents to treat these currently incurable conditions. More broadly the assay can be exploited to study quantitatively in a high‐throughput manner any biochemical process that allows ions to enter vesicles across the lipid bilayer.

## Conflict of interest

The authors declare no conflict of interest.

## Supporting information

As a service to our authors and readers, this journal provides supporting information supplied by the authors. Such materials are peer reviewed and may be re‐organized for online delivery, but are not copy‐edited or typeset. Technical support issues arising from supporting information (other than missing files) should be addressed to the authors.

Supplementary

## References

[1] C. Soto , Nat. Rev. Neurosci. 2003, 4, 49–60. 12511861 10.1038/nrn1007

[2] C. Haass , D. J. Selkoe , Nat. Rev. Mol. Cell Biol. 2007, 8, 101–112. 17245412 10.1038/nrm2101

[3] T. P. J. Knowles , M. Vendruscolo , C. M. Dobson , Nat. Rev. Mol. Cell Biol. 2014, 15, 384–396. 24854788 10.1038/nrm3810

[4] R. Riek , D. S. Eisenberg , Nature 2016, 539, 227–235. 27830791 10.1038/nature20416

[5] I. Benilova , E. Karran , B. De Strooper , Nat. Neurosci. 2012, 15, 349–357. 22286176 10.1038/nn.3028

[6] M. Bucciantini , D. Nosi , M. Forzan , E. Russo , M. Calamai , L. Pieri , L. Formigli , F. Quercioli , S. Soria , F. Pavone , et al., FASEB J. 2012, 26, 818–831. 22071505 10.1096/fj.11-189381

[7] P. Narayan , K. M. Holmström , D. H. Kim , D. J. Whitcomb , M. R. Wilson , P. St. George-Hyslop , N. W. Wood , C. M. Dobson , K. Cho , A. Y. Abramov , et al., Biochemistry 2014, 53, 2442–2453. 24717093 10.1021/bi401606f PMC4004235

[8] S. I. A. Cohen , P. Arosio , J. Presto , F. R. Kurudenkandy , H. Biverstål , L. Dolfe , C. Dunning , X. Yang , B. Frohm , M. Vendruscolo , et al., Nat. Struct. Mol. Biol. 2015, 22, 207–213. 25686087 10.1038/nsmb.2971 PMC4595974

[9] M. Andreasen , N. Lorenzen , D. Otzen , Biochim. Biophys. Acta Biomembr. 2015, 1848, 1897–1907. 10.1016/j.bbamem.2015.01.018 25666871

[10] D. Balchin , M. Hayer-Hartl , F. U. Hartl , Science 2016, 353, aac4354. 10.1126/science.aac4354 27365453

[11] A. Drews , J. Flint , N. Shivji , P. Jönsson , D. Wirthensohn , E. De Genst , C. Vincke , S. Muyldermans , C. M. Dobson , D. Klenerman , Sci. Rep. 2016, 6, 31910. 27553885 10.1038/srep31910 PMC4995397

[12] A. Jan , O. Adolfsson , I. Allaman , A.-L. Buccarello , P. J. Magistretti , A. Pfeifer , A. Muhs , H. A. Lashuel , J. Biol. Chem. 2011, 286, 8585–8596. 21156804 10.1074/jbc.M110.172411 PMC3048741

[13] N. P. Reynolds , A. Soragni , M. Rabe , D. Verdes , E. Liverani , S. Handschin , R. Riek , S. Seeger , J. Am. Chem. Soc. 2011, 133, 19366–19375. 21978222 10.1021/ja2029848

[14] S. I. A. Cohen , S. Linse , L. M. Luheshi , E. Hellstrand , D. A. White , L. Rajah , D. E. Otzen , M. Vendruscolo , C. M. Dobson , T. P. J. Knowles , Proc. Natl. Acad. Sci. USA 2013, 110, 9758–9763. 23703910 10.1073/pnas.1218402110 PMC3683769

[15] H. Chaudhary , A. N. D. Stefanovic , V. Subramaniam , M. M. A. E. Claessens , FEBS Lett. 2014, 588, 4457–4463. 25448986 10.1016/j.febslet.2014.10.016

[16] M. Serra-Batiste , M. Ninot-Pedrosa , M. Bayoumi , M. Gairí , G. Maglia , N. Carulla , Proc. Natl. Acad. Sci. USA 2016, 113, 10866–10871. 27621459 10.1073/pnas.1605104113 PMC5047179

[17] E. Evangelisti , R. Cascella , M. Becatti , G. Marrazza , C. M. Dobson , F. Chiti , M. Stefani , C. Cecchi , Sci. Rep. 2016, 6, 32721. 27619987 10.1038/srep32721 PMC5020652

[18] N. Cremades , S. I. A. Cohen , E. Deas , A. Y. Abramov , A. Y. Chen , A. Orte , M. Sandal , R. W. Clarke , P. Dunne , F. A. Aprile , et al., Cell 2012, 149, 1048–1059. 22632969 10.1016/j.cell.2012.03.037 PMC3383996

[19] M. J. Savage , J. Kalinina , A. Wolfe , K. Tugusheva , R. Korn , T. Cash-Mason , J. W. Maxwell , N. G. Hatcher , S. J. Haugabook , G. Wu , et al., J. Neurosci. 2014, 34, 2884–2897. 24553930 10.1523/JNEUROSCI.1675-13.2014 PMC6608513

[20] M. F. M. Sciacca , S. A. Kotler , J. R. Brender , J. Chen , D. K. Lee , A. Ramamoorthy , Biophys. J. 2012, 103, 702–710. 22947931 10.1016/j.bpj.2012.06.045 PMC3443794

[21] C. Galvagnion , J. W. P. Brown , M. M. Ouberai , P. Flagmeier , M. Vendruscolo , A. K. Buell , E. Sparr , C. M. Dobson , Proc. Natl. Acad. Sci. USA 2016, 113, 7065–7070. 27298346 10.1073/pnas.1601899113 PMC4932957

[22] D. Stamou , C. Duschl , E. Delamarche , H. Vogel , Angew. Chem. Int. Ed. 2003, 42, 5580–5583; 10.1002/anie.200351866 14639720

[23] P. M. Bendix , M. S. Pedersen , D. Stamou , Proc. Natl. Acad. Sci. USA 2009, 106, 12341–12346. 19597158 10.1073/pnas.0903052106 PMC2709668

[24] S. Mathiasen , S. M. Christensen , J. J. Fung , S. G. Rasmussen , J. F. Fay , S. K. Jorgensen , S. Veshaguri , D. L. Farrens , M. Kiskowski , B. Kobilka , et al., Nat. Methods 2014, 11, 931–934. 25086504 10.1038/nmeth.3062 PMC4485457

[25] B. Lohse , P. Y. Bolinger , D. Stamou , J. Am. Chem. Soc. 2008, 130, 14372–14373. 18842043 10.1021/ja805030w

[26] E. Hellstrand , B. Boland , D. M. Walsh , S. Linse , ACS Chem. Neurosci. 2010, 1, 13–18. 22778803 10.1021/cn900015v PMC3368626

[27] P. Narayan , A. Orte , R. W. Clarke , B. Bolognesi , S. Hook , K. A. Ganzinger , S. Meehan , M. R. Wilson , C. M. Dobson , D. Klenerman , Nat. Struct. Mol. Biol. 2012, 19, 79–83. 10.1038/nsmb.2191 PMC4979993 22179788

[28] S. Poon , T. M. Treweek , M. R. Wilson , S. B. Easterbrook-Smith , J. A. Carver , FEBS Lett. 2002, 513, 259–266. 11904161 10.1016/s0014-5793(02)02326-8

[29] G. Paraschiv , C. Vincke , P. Czaplewska , M. Manea , S. Muyldermans , M. Przybylski , J. Mol. Recognit. 2013, 26, 1–9. 23280612 10.1002/jmr.2210

[30] J. J. Yerbury , S. Poon , S. Meehan , B. Thompson , J. R. Kumita , C. M. Dobson , M. R. Wilson , FASEB J. 2007, 21, 2312–2322. 17412999 10.1096/fj.06-7986com

[31] P. Narayan , S. Meehan , J. A. Carver , M. R. Wilson , C. M. Dobson , D. Klenerman , Biochemistry 2012, 51, 9270–9276. 23106396 10.1021/bi301277k PMC4981287

[32] P. Flagmeier , G. Meisl , M. Vendruscolo , T. P. J. Knowles , C. M. Dobson , A. K. Buell , C. Galvagnion , Proc. Natl. Acad. Sci. USA 2016, 113, 10328–10333. 27573854 10.1073/pnas.1604645113 PMC5027465

[33] E. R. Padayachee , H. Zetterberg , E. Portelius , J. Borén , J. L. Molinuevo , N. Andreasen , R. Cukalevski , S. Linse , K. Blennow , U. Andreasson , Brain Res. 2016, 1651, 11–16. 27653981 10.1016/j.brainres.2016.09.022 PMC5090044

[34] M. Hölttä , H. Zetterberg , E. Mirgorodskaya , N. Mattsson , K. Blennow , J. Gobom , PLoS One 2012, 7, e42555. 10.1371/journal.pone.0042555 PMC3412831 22880031

[35] F. Chiti , C. M. Dobson , Annu Rev Biochem. 2006, 75, 333–366. 16756495 10.1146/annurev.biochem.75.101304.123901

