# Supplementary material for: Ultrasensitive Measurement of Ca2+ Influx into Lipid Vesicles Induced by Protein Aggregates
Source: Angew Chem Int Ed Engl. 2017 May 5;56(27):7750–4. doi: 10.1002/anie.201700966 (PMC5615231; doi:10.1002/anie.201700966)
Supplement: Supplementary file 1 — Supplementary [file ANIE-56-7750-s001.pdf]

Angewandte Chemie
Eine Zeitschrift der Gesellschaft Deutscher Chemiker

Supporting Information

Ultrasensitive Measurement of Ca^{2+} Influx into Lipid Vesicles Induced by Protein Aggregates

Patrick Flagmeier[+], Suman De[+], David C. Wirthensohn, Steven F. Lee, Cécile Vincke, Serge Muyldermans, Tuomas P. J. Knowles, Sonia Gandhi, Christopher M. Dobson, and David Klenerman**

anie_201700966_sm_miscellaneous_information.pdf

Abstract: To quantify and characterize the potentially toxic protein aggregates associated with neurodegenerative diseases, a high-throughput assay based on measuring the extent of aggregate-induced Ca^{2+} entry into individual lipid vesicles has been developed. This approach was implemented by tethering vesicles containing a Ca^{2+} sensitive fluorescent dye to a passivated surface and measuring changes in the fluorescence as a result of membrane disruption using total internal reflection microscopy. Picomolar concentrations of Aβ42 oligomers could be observed to induce Ca^{2+} influx, which could be inhibited by the addition of a naturally occurring chaperone and a nanobody designed to bind to the Aβ peptide. We show that the assay can be used to study aggregates from other proteins, such as α-synuclein, and to probe the effects of complex biofluids, such as cerebrospinal fluid, and thus has wide applicability.

DOI: 10.1002/anie.201700966

WILEY-VCH

Experimental Procedures

Preparation and purification of recombinant Aβ42

The recombinant Aβ42 (M1-42) peptide (MDAEFRHDSGYEVHHQKLVFF AEDVGSNKGAIIGLMVGGVVIA), here called Aβ42, was expressed in the *Escherichia coli* BL21 Gold (DE3) strain and purified as described previously with slight modifications[1,2].

Preparation of recombinant Aβ42 for experiments

Solutions of monomeric recombinant Aβ42 were prepared as previously described[1,2] by dissolving the lyophilized Aβ42 peptide in 6 M GuHCl then purifying the protein using a Superdex 75 10/300 GL column (GE Healthcare Bio-Sciences AB SE-751 84 Uppsala, Sweden). The center of the elution peak was collected, and the peptide concentration was determined from the absorbance of the integrated peak area using ε_{280} = 1490 L mol^{-1}cm^{-1}.

Measurement of aggregation kinetics of Aβ42

For kinetic experiments the Aβ42 monomer was diluted with buffer to the desired concentration and supplemented with 20 μM ThT. All samples were prepared in low-binding Eppendorf tubes (Eppendorf AG, Hamburg, Germany) on ice. Each sample was then pipetted into multiple wells of a 96-well half-area, low-binding polyethylene glycol coating plate (Corning 3881, Kennebuck ME, USA) with a clear bottom, at 80 μL per well. The 96-well plate was placed in a plate reader (Fluostar Omega, Fluostar Optima, or Fluostar Galaxy; BMG Labtech, Ortenberg, Germany) and incubated at 37°C under quiescent conditions using the bottom reading mode (440-nm excitation filter, 480-nm emission filter). For each new preparation of protein, the aggregation kinetics were checked by performing reactions at different concentrations of Aβ42.

Conditions for Aβ42 aggregation

Aliquots of monomeric Aβ42 were diluted with buffer to a concentration of 2 μM in low-binding Eppendorf tubes on ice. Individual samples were then pipetted into multiple wells of a 96-well half-area plate (Corning 3881, Kennebuck ME, USA) and the plate was placed into an incubator at 37 °C, under quiescent conditions. Aliquots for measurements of Ca^{2+} influx were then taken at the desired times after the plate was placed in the incubator.

Preparation and Purification of α-synuclein

Recombinant α-synuclein was expressed in the Escherichia coli BL21 Gold (DE3) strain (Stratagene) and purified as described previously[3–5]. In short, the purification involved sonicating the cells, boiling the cell debris, and carrying out ion exchange chromatography using a POROS HQ20 anion exchange column (Applied Biosystems Ltd. Warrington, UK); this step was followed by gel filtration using a HiLoad 26/60 Superdex 75 pg exclusion molecular column (Amersham Biosciences, Sweden). The protein concentration was determined from the absorbance at 275 nm using an extinction coefficient of 5600 M^{-1}cm^{-1}. Protein samples were flash frozen in liquid N_2 and stored at -80°C until use.

WILEY-VCH

Conditions for α-synuclein aggregation

Monomeric α-synuclein was incubated at a concentration of 70 μM in 25 mM Tris-HCl with 100 mM NaCl (pH 7.4) with constant shaking at 200 rpm for 5 h at 37 °C, conditions shown previously to result in the formation of oligomeric species[6].

CSF Sample

The CSF sample was collected from a healthy individual (aged 65 years) by lumbar puncture. Standardized protocols for the collection and storage of CSF (www.neurochem.gu.se/TheAlzAssQCProgram) were followed. In short, the lumbar puncture was performed between 9 a.m. and 12 noon to collect 15 mL of CSF in sterile polypropylene tubes. The sample was divided into 1 mL aliquots that were frozen on dry ice and stored at −80 °C in Sarstedt 2mL tube. The time between sample collection, centrifugation, and freezing was maximum 1 h.

Preparation of the nanobody Nb3 and clusterin

Nb3 was prepared as previously described[7−9]. Briefly, it was recombinantly expressed in *Escherichia coli*[9] and purified using immobilized metal affinity chromatography and size-exclusion chromatography[7]. The concentration was measured by UV absorbance spectroscopy using a molecular extinction coefficient, which was calculated based on the sequence of the protein at 280 nm of 21,555 M^{-1} cm^{-1}. Clusterin was obtained as previously described[10,11], and purified from human serum by IgG affinity chromatography or by affinity chromatography using MAb G7[12].

Optimization of the dye filled vesicle preparation

Initially we screened a series of different dye molecules for this assay. To ensure that we could attach vesicles to the surface and for probing surface coating protocols we used the dye rhodamine (Rh6G) for encapsulation. Thereafter, we tested the Ca^{2+}-sensitive dyes Fluo-4, Fluo-8 and Cal-520 (Stratech Scientific Ltd, Newmarket, UK) and found that we detected the strongest increase in localized fluorescence intensity using the dye Cal-520.

We also examined if the method can be performed using vesicles composed of different lipids and tested 1,2-ditetradecanoyl-sn-glycero-3-phospho-L-serine (*DMPS)* PS(14:0/14:0) (Avanti Polar Lipids, Alabama, USA, Catalogue No. - 840033) and 1-palmitoyl-2-oleoyl-sn-glycero-3-phosphocholine (POPC) PC (16:0/18:1) (Avanti Polar Lipids, Alabama, USA, Catalogue No. - 840033). We selected vesicles composed of POPC to perform our experiments as the bilayer is in the fluid form at room temperature, as previously described[13].

We tested vesicles of varying sizes (50, 100 and 200, 400 nm) and found that all these vesicles can be used in this assay. We probed vesicles containing varying concentrations of incorporated dye,1-100 μM, and found that improved signals can be detected at higher dye concentrations. Higher concentrations of the dye were found to be preferable for focusing of the instrument on samples incubated in L15 medium or samples that did not induce Ca^{2+} influx. However, the incorporation of higher concentrations of dye molecules into the vesicles resulted in the surrounding solution containing a high concentration of free dye, we therefore performed size exclusion chromatography in order to remove free dye molecules from the surrounding solution. We tested both non-purified and purified vesicle samples and found that we observed considerably less background signal using purified vesicles.

WILEY-VCH

Finally, based on these optimizations and our calculations (see Supporting Information Note 1 and 2, Supporting Information Fig. 1) we performed our experiments using purified vesicles composed of POPC with an average size of 200 nm containing Cal-520 at a concentration of 100 µM.

Preparation and purification of dye filled vesicles

Phospholipids 16:0-18:1 PC (catalogue no - 850457) and biotinylated lipids 18:1-12:0 Biotin PC (catalogue no - 860563) were purchased from Avanti Polar Lipids (Alabama, USA) in the form of powder and chloroform solutions respectively. For lipids bought as powder, chloroform stock solutions (1 mg/mL) were prepared and stored at -20°C. Then stock solutions were mixed such that the ratio between 16:0-18:1 PC and 18:1-12:0 biotin PC was 100:1 lipid. The chloroform was then removed under vacuum in a desiccator overnight. The samples were then dissolved in HEPES buffer (pH 6.5) with 100 µM Cal-520 and five freeze-and-thaw cycles were performed using dry ice and a water bath. The solution was passed at least 10 times through an extruder (Avanti Polar Lipids, Alabama, USA) with a membrane of an appropriate size cut off to obtain vesicles of the desired size, The size of the vesicles was determined using a Zetasizer (Zetasizer Nano ZSP, Malvern Instruments, Malvern, UK).

To separate non-incorporated dye molecules from the vesicles, size-exclusion chromatography was performed in buffer using a Superdex™ 200 Increase 10/300 GL column attached to an AKTA pure system (GE Life Sciences) with a flow rate of 0.5 mL/min (Supporting Information Fig. 3).

Preparation of PEGylated slides and immobilization of single vesicles

Initially we screened a variety of surface treatment protocols[13–18] and for our experiments we optimized and followed a previously described protocol[18] with slight modifications to perform the actual experiments. Borosilicate glass coverslides (VWR International, 22x22 mm, product number 63 1-0122) were cleaned by sonicating in 2% (v/v) Hellmanex III (Hellma GmbH & Co. KG, Müllheim, Germany) in milliQ water for 10 min followed by sonicating twice in milliQ water and in methanol for 10 min each and then in water again for 10 more minutes. The glass slides were dried under a nitrogen stream, and plasma-etched using an argon plasma cleaner (PDC-002, Harrick Plasma, Ithaca, NY) for 20 minutes to remove any fluorescent impurities. Frame-Seal incubation chambers (9x9mm^2, Biorad, Hercules, CA, product number SLF-0601) were affixed to the glass slides and 50 µL of a mixture of 100:1 PLL-g-PEG (SuSoS AG, Dübendorf, Switzerland) and PLL-g-PEG biotin (SuSoS AG, Dübendorf, Switzerland) (1 g/L) in reaction buffer (50 mM Hepes, pH 6.5) was added to the coverslide inside of the chamber and incubated for 30 min. Then the coverslides were washed 3 times with filtered reaction buffer. 50 µL of a solution of 0.1 mg/mL Neutravidin (ThermoScientific, Rockford, IL 61105, USA) in reaction buffer was added to the coverslide and incubated for 15 min, and washed 3 times with reaction buffer. Then, 50 µL of the solution of purified vesicles was added to the coverslide and incubated for 30 min before washing carefully at least 5 times with reaction buffer.

Imaging using Total Internal Reflection Fluorescence Microscope

Imaging was performed using a homebuilt Total Internal Reflection Fluorescence Microscope (TIRFM) based on an inverted Olympus IX-71 microscope. This imaging mode restricts the detected fluorescence signal to within 100-150 nm from the glass-water interface. A 488 nm laser (Toptica, iBeam smart, 200 mW, Munich, Germany) was used to excite the sample. The expanded and collimated laser beam was focused using two Plano-convex lens onto the back-focal plane of the 60X, 1.49NA oil immersion objective lens (APON60XO TIRF, Olympus,

product number N2709400) to a spot of adjustable diameter. The fluorescence signal was collected by the same objective and was separated from the excitation beam by a dichroic (Di01-R405/488/561/635, Semrock). The emitted light was passed through an appropriate set of filters (BLP01-488R, Semrock and FF01-520/44-25, Semrock) (Figure S14). The fluorescence signal was then passed through a 2.5x beam expander and imaged onto a 512 × 512 pixel EMCCD camera (Photometrics Evolve, E VO-512-M-FW- 16-AC-110). Images were acquired with a 488nm laser (~10 W/cm^2) for 50 frames with a scan speed of 20 Hz and bit depth of 16 bits. Each pixel corresponds to 100 nm. All the measurements were carried out under ambient conditions (T=295K). The open source microscopy manager software Micro Manager 1.4 was used to control the microscope hardware and image acquisition[19,20].

Performing the Ca^{2+} influx assay using TIRFM

Single vesicles tethered to PLL-PEG coated borosilicate glass coverslides (VWR International, 22x22 mm, product number 63 1-0122) were placed on an oil immersion objective mounted on an inverted Olympus IX-71 microscope. Each coverslide was affixed at Frame-Seal incubation chambers and was incubated with 50 µL of HEPES buffer of pH 6.5. Just before the imaging, the HEPES buffer was replaced with 50 µL Ca^{2+} containing buffer solution L-15. 16 (4×4) images of the coverslide were recorded under three different conditions (background, in the presence of Aβ42 and after addition of ionomycin (Cambridge Bioscience Ltd, Cambridge, UK), respectively). The distance between each field of view was set to 100 µm, and was automated (bean-shell script, Micromanager) to avoid any user bias (Figure S3). After each measurement the script allowed the stage (Prior H117, Rockland, MA, USA) to move the field of view back to the start position such that identical fields of view could be acquired for the three different conditions. We screened surface treatment protocols, PEG: biotin-PEG ratios, vesicle size, different encapsulate Ca^{2+} binding dyes and their concentrations to maximize the sensitivity of this assay.

Images of the background were acquired in the presence of L15 buffer. For each field of view 50 images were taken with an exposure time of 50 ms. Thereafter, 50 µL of the aggregation reaction, diluted to a concentration of twice the targeted value, was added and incubated for 10 min. Importantly we made sure that the glass coverslides were not moved during the addition of samples and then images were recorded. Next, 10 µL of a solution containing 1 mg/mL of ionomycin (Cambridge Bioscience Ltd, Cambridge, UK) was added and incubated for 5 min and subsequently images of Ca^{2+} saturated single vesicles in the same fields of view were acquired.

Experiments with recombinant Aβ42 in CSF

To study the influence of the presence of a complex environment on the Ca^{2+} influx, we have taken samples of recombinant Aβ42 aggregation reactions corresponding to t_2 and serially diluted it in the CSF to measure the concentration dependence of the Ca^{2+} influx. Firstly, we imaged the coverslides in presence of 15 µL of L15 buffer. Then aliquots of recombinant Aβ42 were diluted in 15 µL of CSF which was added to the coverslides and incubated for 10 min before images were acquired as described previously. Thereafter, we added ionomycin to the sample and imaged the identical fields of view using automatic stage movement to determine the Ca^{2+} influx.

Data analysis and quantification of the extent of Ca^{2+} influx

The recorded images were analyzed using ImageJ[21,22] to determine the fluorescence intensity of each spot under the three different conditions, namely background ($F_{background}$), in the presence of an aggregation mixture

($F_{aggregate}$), and after the addition of ionomycin ($F_{Ionomycin}$). The relative influx of Ca^{2+} into an individual vesicle due to aggregates of Aβ42 peptide was then determined using the following equation:

$$Ca^{2+}\text{influx} = \frac{F_{aggregate} - F_{background}}{F_{Ionomycin} - F_{blank}} \qquad (1)$$

The average degree of Ca^{2+} influx was calculated by averaging the Ca^{2+} influx into individual vesicles.

Results and Discussion

Supporting Information Note 1: Calculation of the concentration of an individual dye molecule entering into a vesicle

The volume of a single vesicle with a diameter d can be calculated using equation (1).

$$V_{Vesicles} = \frac{4}{3}\pi\left(\frac{d}{2}\right)^3 \qquad (1)$$

We used vesicles with diameters of approximately 200 nm, which have a volume of 4.186 x 10^{-18} L. For a single molecule, the number of moles can be calculated using Avogadro's number ($N_{avogadro}$ = 6.023 x 10^{23}). Using this value we can determine the concentration of a single molecule that enters a vesicle using equation (2).

$$[Single\ molecule]_{in\ Vesicle} = \left(V_{Vesicle} \times N_{Avogadro}\right)^{-1} \qquad (2)$$

Thus, the concentration of a single molecule entering a vesicle with a diameter of 200 nm is 396 nM (Supporting Information Fig. 1).

Supporting Information Note 2: Rationalization for using vesicles with a diameter of 200nm

The effectiveness of our single vesicle assay is primarily determined by two parameters with different dependencies on the size of a vesicle – (i) high dynamic range and (ii) high sensitivity (Supporting Information Fig. 1).

High dynamic range is the capacity to detect differences in fluorescence intensity over a range of varying amounts of Ca^{2+} influx within individual vesicles, without reaching saturation. The dynamic range of the assay described here is related to the maximum amount of measurable Ca^{2+} influx (*e.g.* when all dye molecules within a single vesicle are saturated with Ca^{2+}), which is directly proportional to the volume of a vesicle. For example, using vesicles with a larger volume enables the encapsulation of more Cal-520 dye molecules, therefore reaching saturation of the maximum fluorescence intensity at larger amounts of Ca^{2+} influx compared to a vesicle of a smaller volume with less Cal-520 dye molecules incorporated.

High sensitivity means the ability to detect low levels of Ca^{2+} influxes within an individual vesicle. The detection sensitivity for Ca^{2+} influx is inversely proportional to the volume of each vesicle. For example, the effective concentration of a single Ca^{2+} ion within a vesicle increases with a decrease of the volume of the vesicle. Hence, a small volume of a vesicle increases the sensitivity of the detection of our assay because smaller changes in the absolute number of Ca^{2+} ions relate to a larger change in their effective concentration.

WILEY-VCH

More specifically, with the aim of maximizing the sensitivity of our assay we designed our assay such that the effective concentration of a single Ca^{2+} ion entering an individual vesicle is in the range of the dissociation constant of the dye Cal-520 (K_d - 320 nM). We determined, using equation (1), that a vesicle with a diameter of approximately 215 nm fulfils the requirement to achieve our target that a single Ca^{2+} entering such a vesicle would have an effective concentration of 320 nM. As outlined above for vesicles with a diameter of 215 nm or less, a single ion entering the vesicle will result in an ion concentration slightly above the K_d of Cal-520. The result is an increase of the fraction of the dye bound to Ca^{2+}, which can be detected using fluorescence microscopy methods. Therefore, in order to achieve the maximum sensitivity with a wide dynamic range of the assay presented in this work, we fabricated vesicles with a diameter of approximately 200 nm.

WILEY-VCH

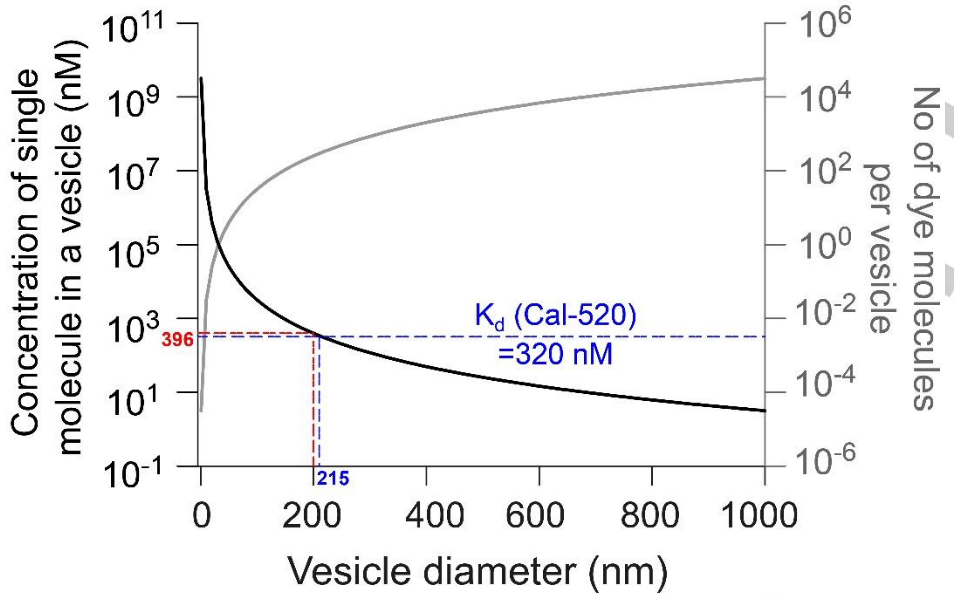

Supporting Information Figure S1: **Design of the assay to achieve an optimum sensitivity and wide dynamic range.** The effective concentration of a single Ca^{2+} (black line) and the number of encapsulated dye molecules (grey line) in a vesicle as a function of its diameter. The diameter of a vesicle (215 nm) such that a single Ca^{2+} entering the vesicle has an effective concentration close to the K_d of the Cal-520 dye (320 nM) is highlighted in blue. The effective concentration of a single Ca^{2+} entering a vesicle with a diameter of 200 nm, as used in the present work, is 396 nM and shown in red.

WILEY

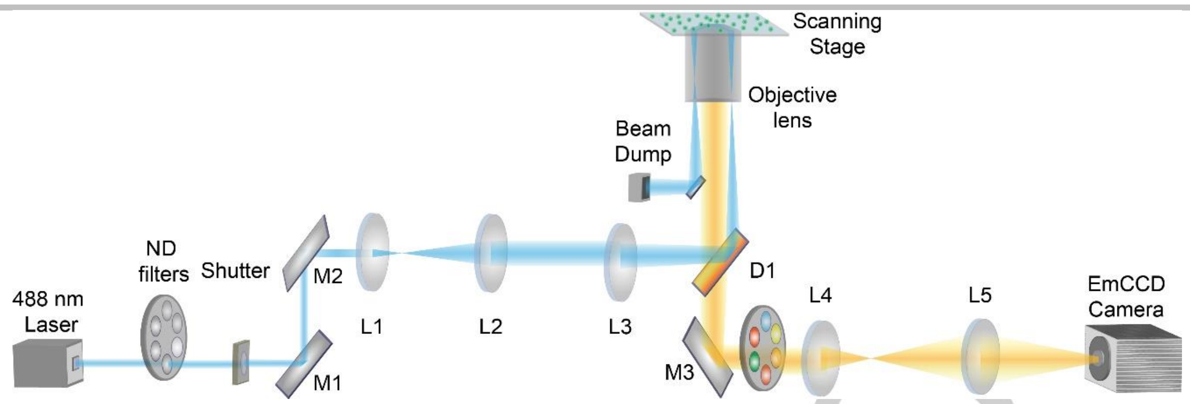

Supporting Information Figure S2: Schematic of the total internal reflection microscopy (TIRFM) set-up. A 488 nm laser line was used to excite the sample. The power was controlled by a combination of neutral-density filters (ND filters). The exposure time is controlled automatically with a mechanical shutter. The laser beam were expanded and collimated with the telescopes L1 and L2. Laser beams were aligned using combinations of mirrors M1-M2. An ocular lens L3 and a dichroic beam splitter D1 were used to focus the collimated beam on the back aperture of the objective for wide field illumination. The emitted fluorescent light was collected by the same objective. The TIRF mode was achieved by of axis movement of the beam at the glass-water interface and the resulting fluorescence was focused using a combination of L4 and L5 onto the electron-multiplying Charge Coupled Device (EMCCD) chip.

WILEY-VCH

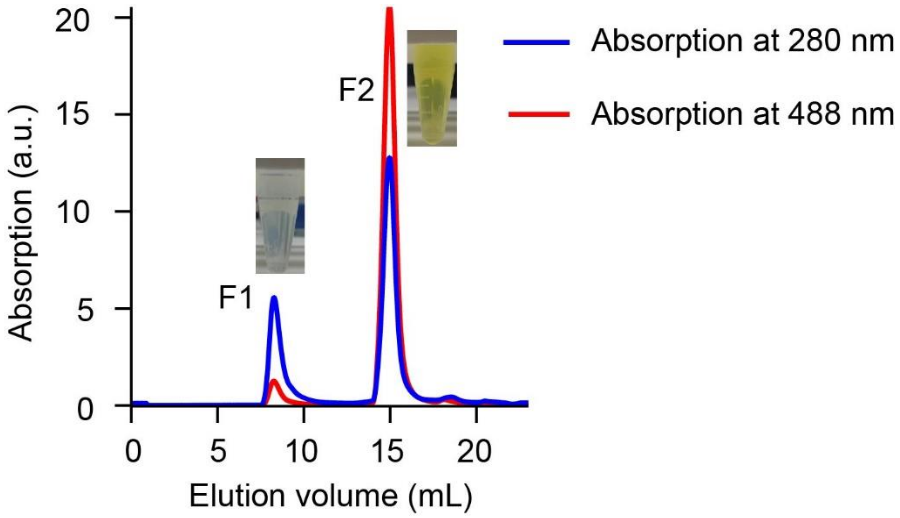

Supporting Information Figure S3: Separation of dye filled lipid vesicles from non-incorporated free dye. Lipid vesicles composed of 16:0-18:1 PC and 18:1-12:0 Biotin PC (ratio 100:1) were prepared in HEPES buffer (pH 6.5) with 100 µM Cal-520 dye by five freeze-and-thaw cycles and extrusion. Size-exclusion chromatography was performed in HEPES buffer (pH 6.5) using a Superdex™ 200 Increase 10/300 GL column attached to an ÄKTA pure system (GE Life Sciences) and the absorption was monitored at 280 nm (blue) and 488 nm (red). Photographs of the fractions (F1, vesicle fraction) and F2 (lipid, free dye) are shown.

WILEY

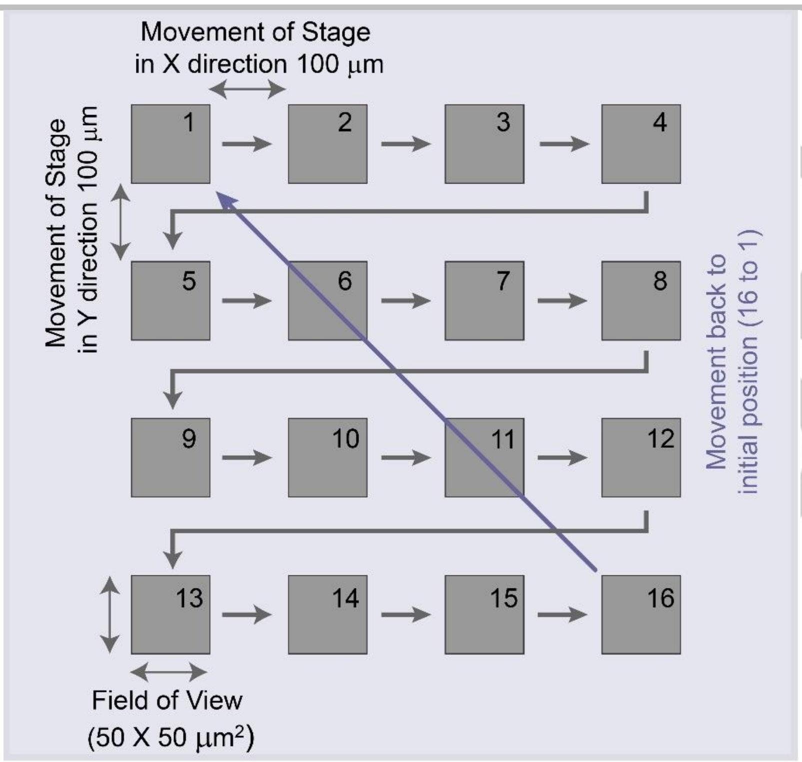

Supporting Information Figure S4: **Automatic stage movement to enable a high-throughput single-vesicle assay.** To calculate the percentage of Ca^{2+} influx in individual vesicles, we compared the same vesicles under three different conditions - the presence of Ca^{2+} buffer, after the addition of a sample and then after the addition of ionomycin. Using a bean-shell based in-house written script, we scanned and imaged 16 (4x4) identical fields of view (dark grey squares) using automatic stage movement. Each of the fields of view was 50 µm x 50 µm and the distance between two consecutive fields is 100 µm. After the last image (16), the stage automatically moves to the initial position of the slide (1, enabling us to scan identical fields of view. Our script allowed us to choose the number of areas to be imaged as well as the distance between them.

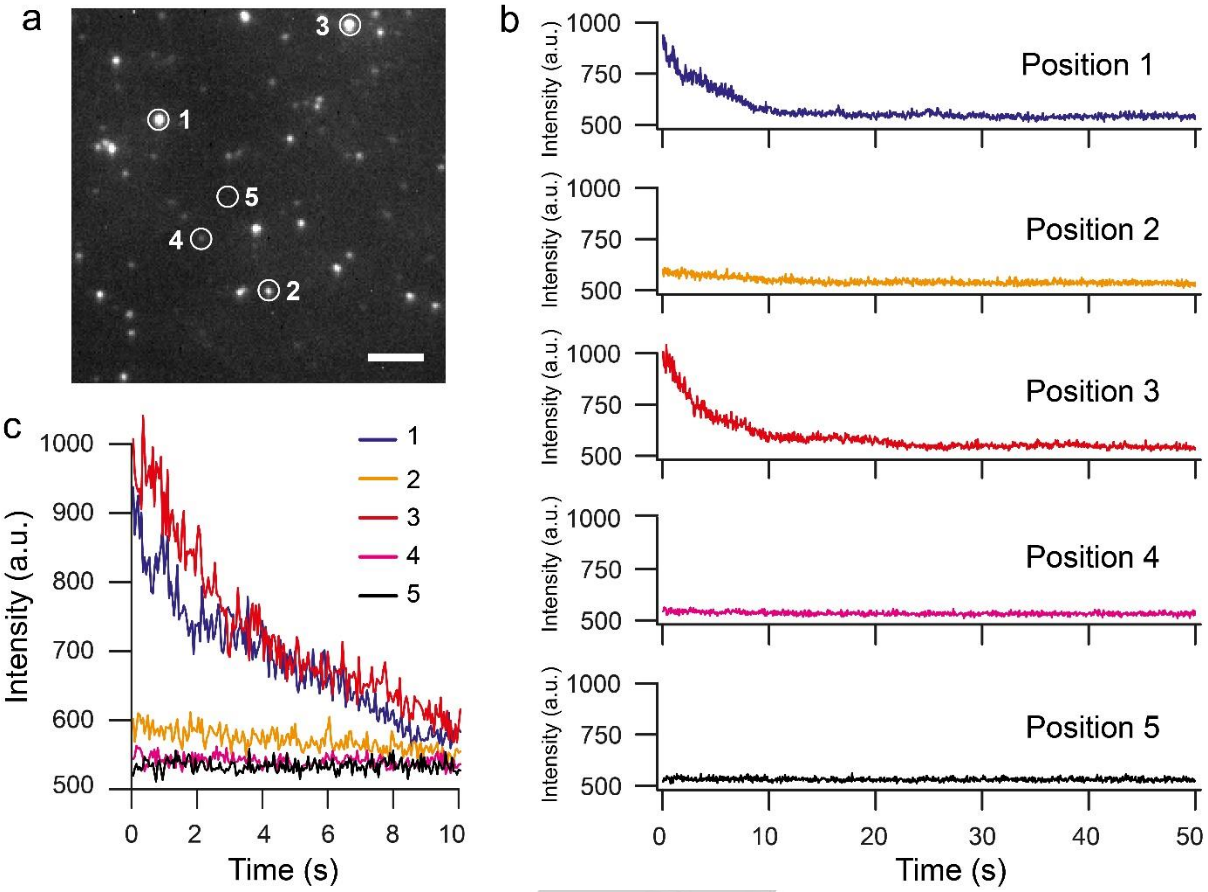

Supporting Information Figure S5: **Photobleaching of the Cal-520 dye molecules encapsulated into individual vesicles. (a)** Surface-tethered single vesicles with a diameter of 200 nm containing the dye Cal-520 appear as bright spots under illumination with 488-nm laser (at higher power; 100 W/cm^2). The image was averaged over 1000 frames of 50 ms exposure time. The circles indicate spots that correspond to the photobleaching traces in b and c (The scale bar represents 3 µm) **(b)** Example of typical photobleaching traces of vesicles containing Cal-520 dye molecules (Position 1-4) and the background (5). **(c)** Zoom of the early times of the photobleaching traces shown in b. Continuous bleaching of localized bright spots indicates the presence of multiple dye molecules within the vesicles. The different averaged intensities as well as the different photobleaching times show the diverse encapsulation efficiencies of individual vesicles.

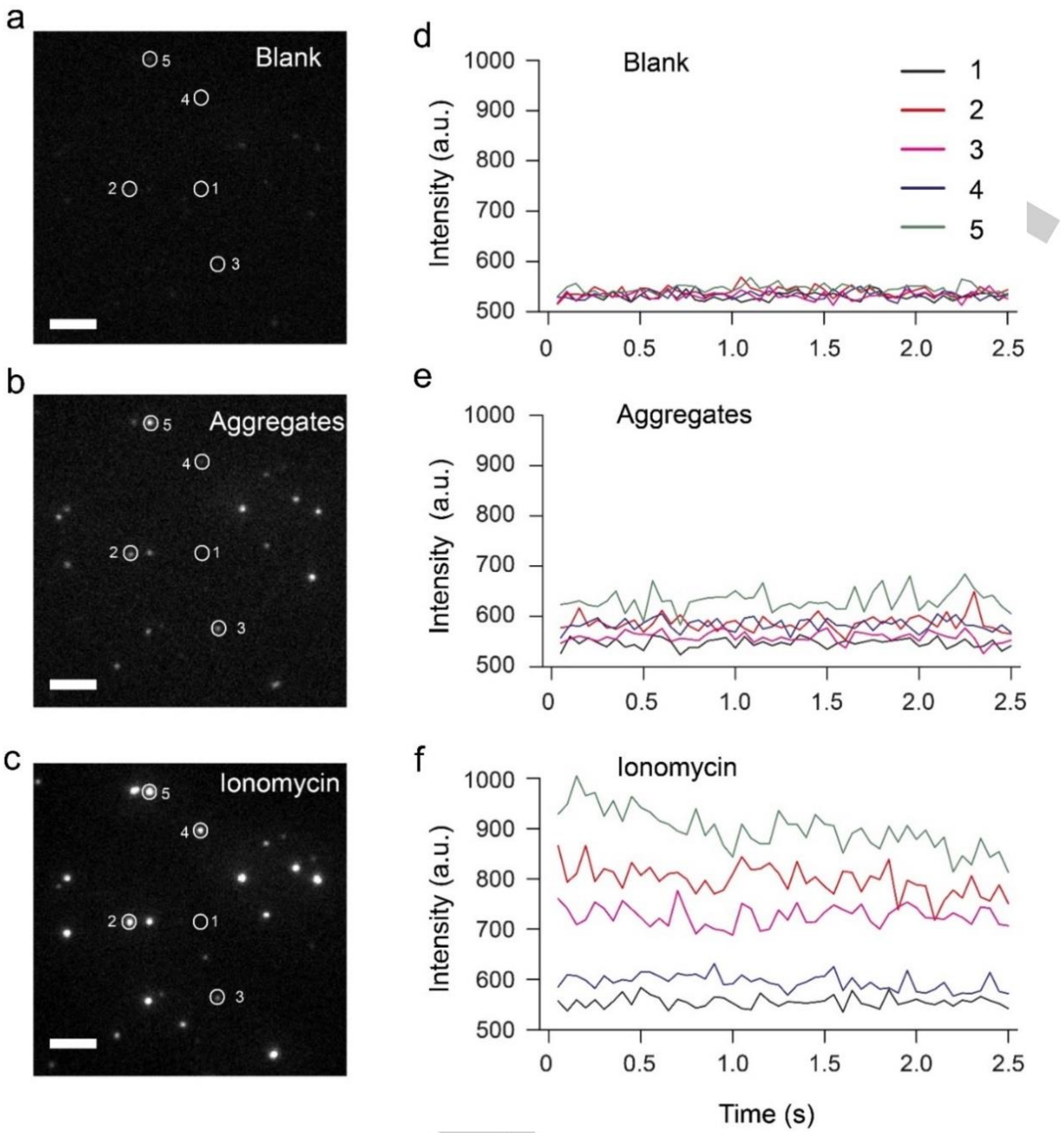

Supporting Information Figure S6: **Intensity traces of single vesicles under different conditions.** 15x15 µm^2 TIRF image of the coverslide with surface tethered vesicles containing multiple Cal-520 dye molecules incubated with **(a)** L15 buffer only , **(b)** Aβ42 aggregates and **(c)** ionomycin. Images were acquired using 10 W/cm^2 power and averaged over 50 frames with 50 ms exposure time. Each bright localized spot corresponds to a vesicle filled with dye. The circles indicate the spots that were used for intensity profiles. The scale bar represents 3 µm. Intensity traces of background (1) and four representative vesicles containing the Cal-520 dye (2-5) are shown in the presence of **(d)** only L-15 buffer, **(e)** Aβ42 aggregates and **(f)** ionomycin. The intensity of the dye molecules encapsulated within the vesicles does not notably change for most of the vesicles in the power regime and the time scales (2.5 s) that were used in these experiments.

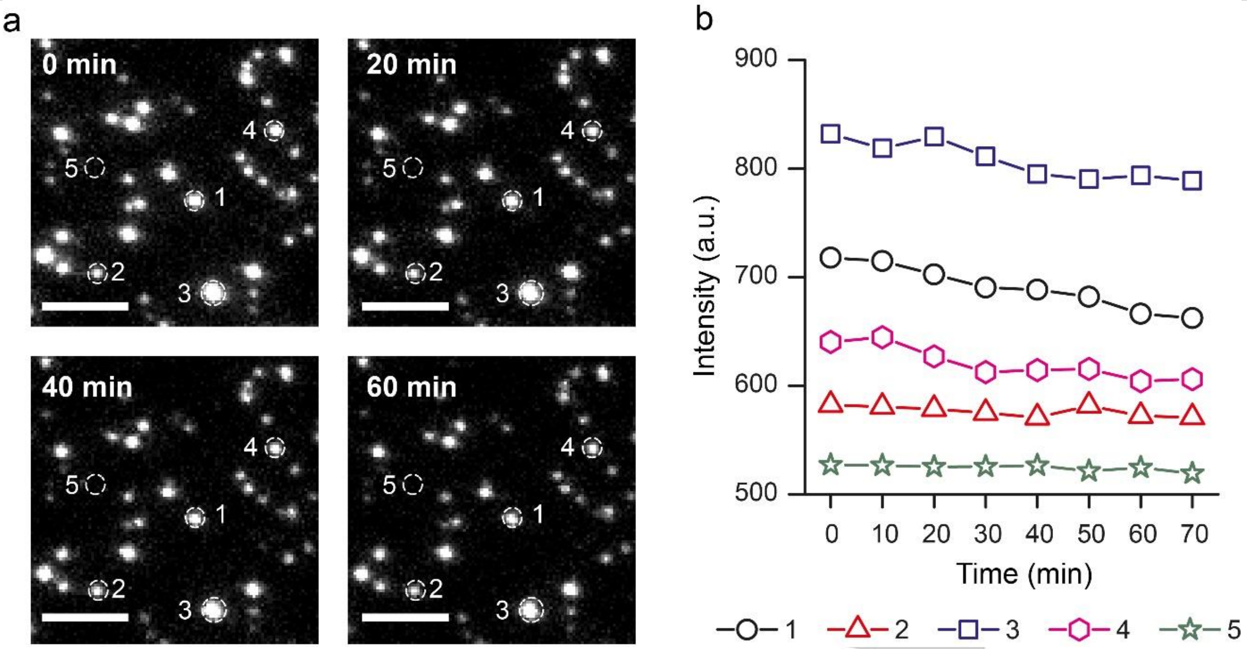

Supporting Information Figure S7: **Photo-stability of Cal-520 dye filled single vesicles in the presence of Ca^{2+}. (a)** The coverslide with surface-tethered vesicles containing Cal-520 dye molecules was incubated in the presence of Ca^{2+} containing phenol free L15 buffer and imaged at 0, 20, 40 and 60 minutes of incubation (the scale bar represents 3 µm). The circles indicate the spots that were used for intensity profiles. We have seen photobleaching of dye molecules in individual vesicles but the vesicles show structural integrity over the experimental time scales. **(b)** Typical intensity traces for vesicles containing Cal-520 dye molecules (Position 1-4) and the background (Position 5). Each point corresponds to the average of 10 frames with an exposure time of 50 ms. The measurements were performed at high power (at 100 W/cm^2) to enable the vesicles to be detected.

WILEY

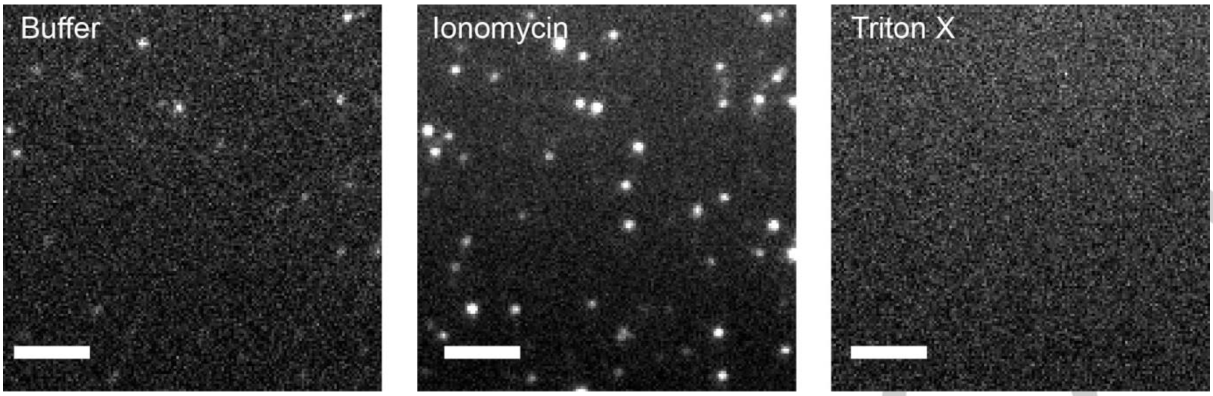

Supporting Information Figure S8: **Detectable fluorescence of surface tethered vesicles containing Cal-520 dye molecules incubating with solutions containing different membrane disrupting reagents.** The surface tethered vesicles containing Cal-520 dye molecules can be detected as bright localized spots in the presence of Ca^{2+} containing buffer under 488-nm illumination. The addition of ionomycin significantly increases the fluorescence intensity, demonstrating that ionomycin allows Ca^{2+} to enter the vesicles. After the addition of Triton X no localized fluorescence was detected, which suggests that the addition of the detergent disrupts the membrane. The scale bar represents 5 µm. These experiments were performed at a higher laser power (at 100 W/cm^2) to allow the detection of Cal-520 dye molecules not bound to Calcium.

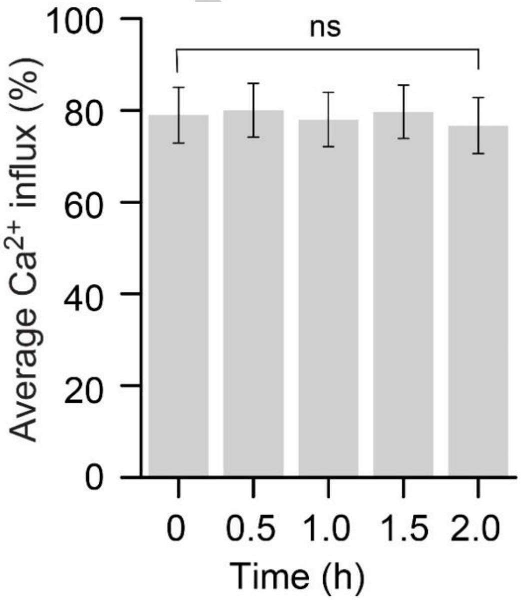

Supporting Information Figure S9: **The Ca^{2+} influx caused by an Aβ42 sample diluted by a factor of 200 remains unchanged over a time period of 2 h of incubation at the diluted concentration.** Aliquots were taken from an aggregation reaction of Aβ42 at a time-point corresponding to t_2 (Figure 2a) and diluted by a factor of 200 to a concentration of 10 nM (monomer equivalents). The diluted sample was incubated at room temperature and at the indicated time points (0, 0.5, 1, 1.5, and 2 h) after dilution of the sample, the ability of the oligomers to permeate the membrane was tested. We found no significant change in their ability to permeate the membrane and induce Ca^{2+} influx.

WILEY-VCH

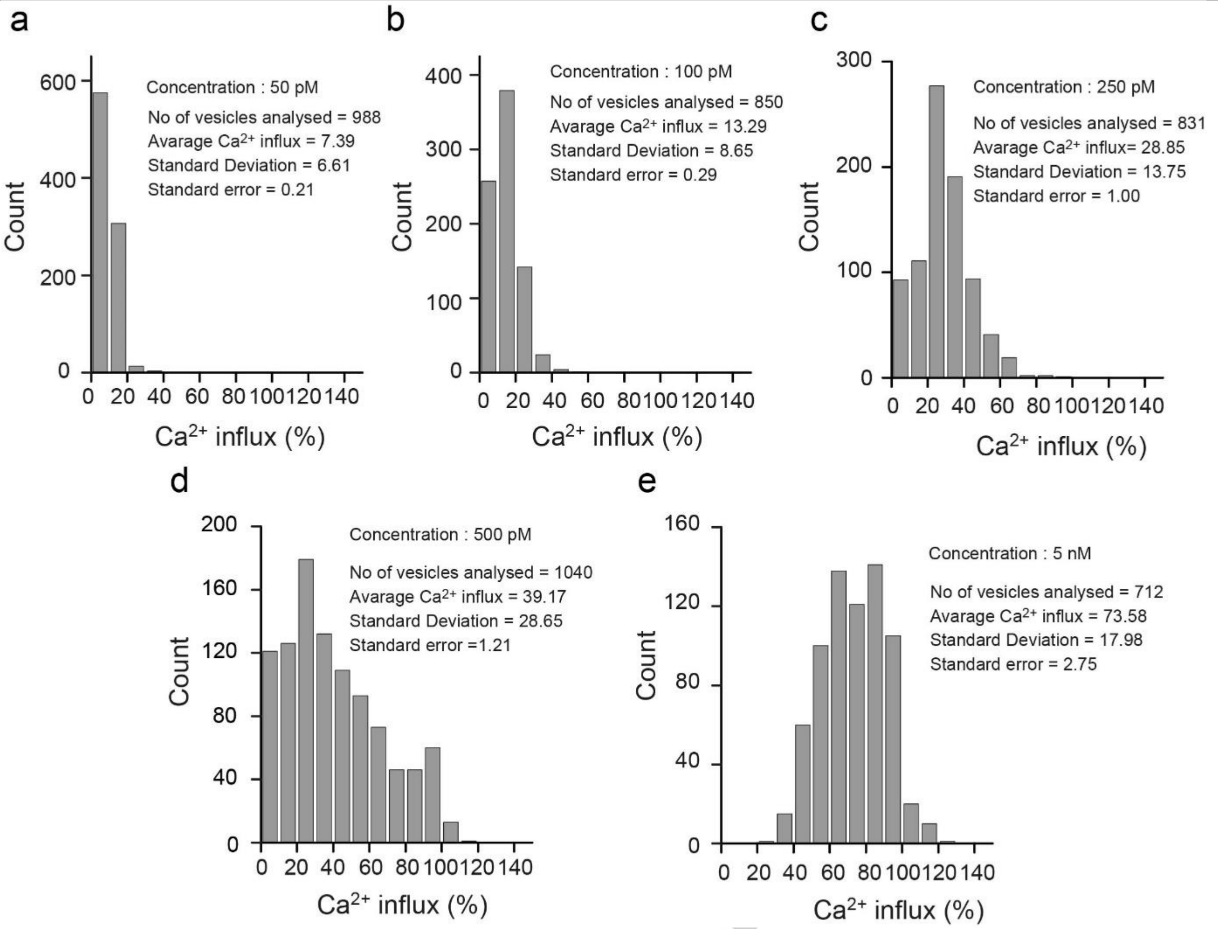

Supporting Information Figure S10**: The distribution of the percentage of Ca^{2+} influx into single vesicles depending on the concentration of Aβ42 aggregates.** Histograms showing the percentage of Ca^{2+} influx into individual vesicles after the addition of aliquots taken of an aggregation reaction of 2 µM monomeric Aβ42 at a time point corresponding to t_2. An aliquot was taken, diluted to the desired concentration and the Ca^{2+} influx was determined. The Ca^{2+} influx was calculated for individual vesicles and binned using 10% steps. The number of vesicles detected, the mean percentage of Ca^{2+} influx, the standard deviation and the standard error of mean are shown for each concentration of Aβ42.

WILEY

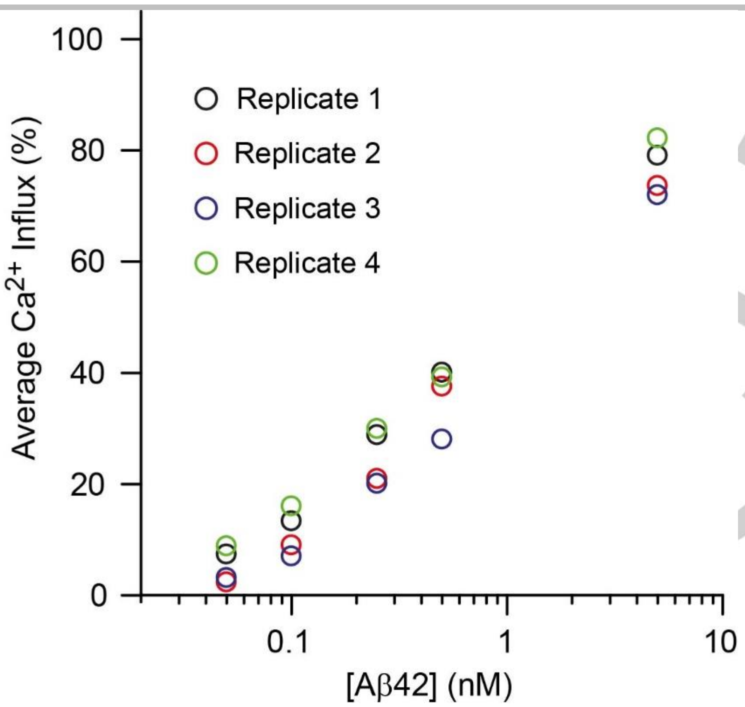

***Supporting Information Figure S11*: Reproducibility of the dependence of the percentage of Ca^{2+} influx on the Aβ42 concentration.** Monomeric Aβ42 was incubated at a concentration of 2 μM and an aliquot was taken at a time point corresponding to t_2. Aliquots were taken and diluted to the desired total Aβ42 monomer concentration. The concentration dependence of the Ca^{2+} influx is shown for four independent experiments (replicates) for the concentration range from 50 pM to 5 nM for which we observed a linear increase in the Ca^{2+} influx. The mean of the percentage of Ca^{2+} influx of these replicates is shown as the average Ca^{2+} influx in Fig. 2d.

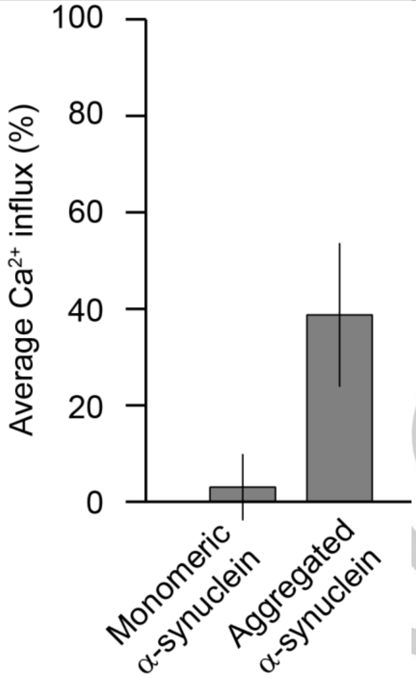

Supporting Information Figure S12: **Ca^{2+} influx induced by α-synuclein aggregates.** Monomeric α-synuclein was incubated at a concentration of 70 μM in 25 mM Tris-HCl with 100 mM NaCl (pH 7.4) for 5 h at 37 °C with constant shaking at 200 rpm. Both for the monomeric protein (Monomeric α-synuclein) and the aggregation mixture (Aggregated α-synuclein), aliquots were taken, diluted to a concentration of 5 nM monomeric α-synuclein and the percentage of Ca^{2+} influx was determined. The detection of increased Ca^{2+} influx upon the addition of α-synuclein aggregates indicates that the Ca^{2+} influx is due to the presence of the latter.

WILEY-VCH

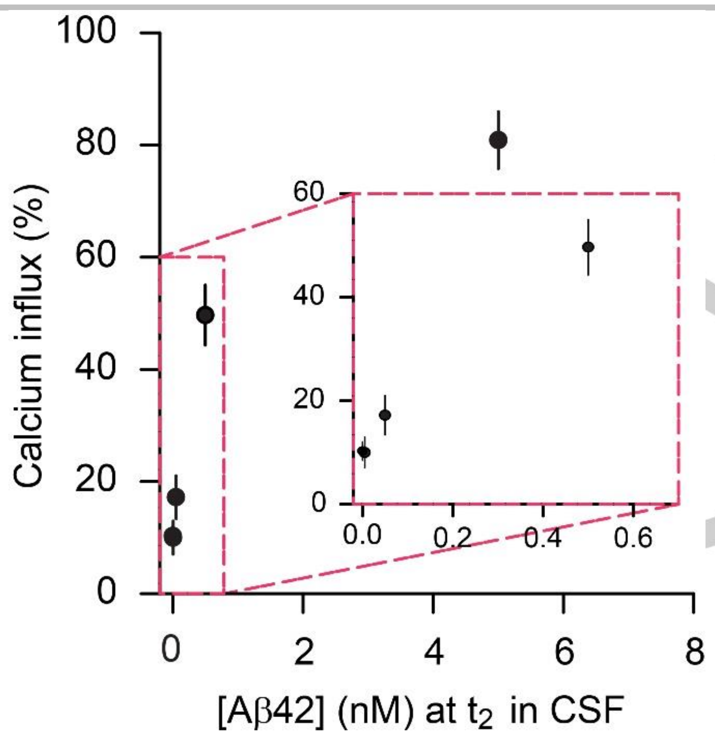

Supporting Information Figure S13: **Recombinant Aβ42 aggregates induce Ca^{2+} influx in the presence of a complex biofluid.** Monomeric Aβ42 was incubated at a concentration of 2 µM under quiescent conditions at 37 °C and an aliquot was taken at a time point corresponding to t_2. The aliquot was diluted to the indicated concentrations of total Aβ42 monomer equivalent in a single sample of control human CSF and the Ca^{2+} influx measured. The insert is a zoom from 0 to 0.7 nM.

WILEY-VCH

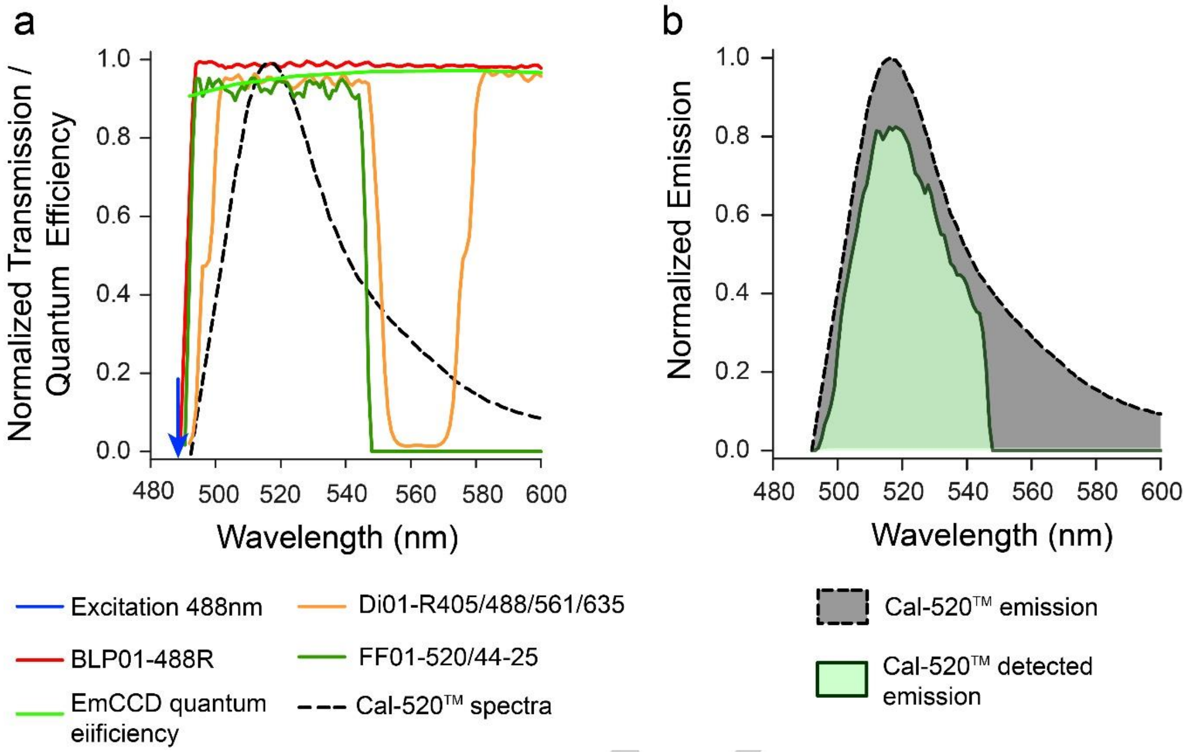

Supporting Information Figure S14: **Estimation of the detected photons from the emission of Cal-520 dye molecules. (a)** Laser excitation at 488 nm and transmission efficiency of dichroic beam splitter (Di01-405/488/561/635), long pass filter (BLP01-488), and band pass filter (FF01-520/44-25) as well as the normalized Quantum efficiency for an EMCCD Photometrics Evolve (EVO-512-M) are shown. **(b)** The emission profile of Cal-520 molecules measured with a Fluorimeter (black dashed line, grey area) and the calculated Cal-520 emission profile (green) with using the BLP01-488 and FF01-520/44-25 filters are shown. We calculated the emission efficiency (62%) by taking into account the efficiency of the dichroic beam splitter, the filters and the efficiency of the detector.

WILEY

References

[1] D. M. Walsh, E. Thulin, A. M. Minogue, N. Gustavsson, E. Pang, D. B. Teplow, S. Linse, *FEBS J.* **2009**, *276*, 1266–1281.

[2] E. Hellstrand, B. Boland, D. M. Walsh, S. Linse, *ACS Chem. Neurosci.* **2010**, *1*, 13–18.

[3] A. K. Buell, C. Galvagnion, R. Gaspar, E. Sparr, M. Vendruscolo, T. P. J. Knowles, S. Linse, C. M. Dobson, *Proc. Natl. Acad. Sci. U. S. A.* **2014**, *111*, 7671–7676.

[4] C. Galvagnion, A. K. Buell, G. Meisl, T. C. T. Michaels, M. Vendruscolo, T. P. J. Knowles, C. M. Dobson, *Nat. Chem. Biol.* **2015**, *11*, 229–234.

[5] P. Flagmeier, G. Meisl, M. Vendruscolo, T. P. J. Knowles, C. M. Dobson, A. K. Buell, C. Galvagnion, *Proc. Natl. Acad. Sci.* **2016**, *113*, 10328–10333.

[6] N. Cremades, S. I. A. Cohen, E. Deas, A. Y. Abramov, A. Y. Chen, A. Orte, M. Sandal, R. W. Clarke, P. Dunne, F. A. Aprile, et al., *Cell* **2012**, *149*, 1048–1059.

[7] K. E. Conrath, M. Lauwereys, M. Galleni, A. Matagne, J.-M. Frere, J. Kinne, L. Wyns, S. Muyldermans, *Antimicrob. Agents Chemother.* **2001**, *45*, 2807–2812.

[8] A. Drews, J. Flint, N. Shivji, P. Jönsson, D. Wirthensohn, E. De Genst, C. Vincke, S. Muyldermans, C. Dobson, D. Klenerman, *Sci. Rep.* **2016**, *6*, 31910.

[9] G. Paraschiv, C. Vincke, P. Czaplewska, M. Manea, S. Muyldermans, M. Przybylski, *J. Mol. Recognit.* **2013**, *26*, 1–9.

[10] M. R. Wilson, S. B. Easterbrook-Smith, *Biochim. Biophys. Acta (BBA)/Protein Struct. Mol.* **1992**, *1159*, 319–326.

[11] S. Poon, T. M. Treweek, M. R. Wilson, S. B. Easterbrook-Smith, J. A. Carver, *FEBS Lett.* **2002**, *513*, 259–266.

[12] M. R. Wilson, P. J. Roeth, S. B. Easterbrook-Smith, *Biochem. Biophys. Res. Commun.* **1991**, *177*, 985–990.

[13] D. Stamou, C. Duschl, E. Delamarche, H. Vogel, *Angew. Chemie - Int. Ed.* **2003**, *42*, 5580–5583.

[14] B. Liu, A. Mazouchi, C. C. Gradinaru, *J. Phys. Chem. B* **2010**, *114*, 15191–15198.

[15] M. Kyoung, Y. Zhang, J. Diao, S. Chu, A. T. Brunger, *Nat. Protoc.* **2013**, *8*, 1–16.

[16] S. Kilic, A. L. Bachmann, L. C. Bryan, B. Fierz, *Nat. Commun.* **2015**, *6*, 7313.

[17] Christensen, Bolinger, Hatzakis, Mortensen, Stamou, *Nat. Nanotechnol.* **2011**, *7*, 51–55.

[18] S. Mathiasen, S. M. Christensen, J. J. Fung, S. G. Rasmussen, J. F. Fay, S. K. Jorgensen, S. Veshaguri, D. L. Farrens, M. Kiskowski, B. Kobilka, et al., *Nat Methods* **2014**, *11*, 931–934.

[19] A. Edelstein, N. Amodaj, K. Hoover, R. Vale, N. Stuurman, in *Curr. Protoc. Mol. Biol.*, John Wiley & Sons, Inc., **2001**.

[20] A. D. Edelstein, M. A. Tsuchida, N. Amodaj, H. Pinkard, R. D. Vale, N. Stuurman, *J. Biol. Methods* **2014**, *1*, 1–10.

[21] J. Schindelin, I. Arganda-Carreras, E. Frise, V. Kaynig, M. Longair, T. Pietzsch, S. Preibisch, C. Rueden, S. Saalfeld, B. Schmid, et al., *Nat. Methods* **2012**, *9*, 676–682.

[22] C. a Schneider, W. S. Rasband, K. W. Eliceiri, *Nat. Methods* **2012**, *9*, 671–675.

Author Contributions

P.F., S.D., T.P.J.K., C.M.D and D.K. designed the study based on DK's original concept, P.F. and S.D. performed the optimization of the method, experiments and data analysis. C.V. and S.M. contributed the nanobody Nb3, S.G. contributed sample of CSF, P.F., S.D., C.M.D. and D.K. interpreted the data. P.F., S.D., C.M.D and D.K. wrote the manuscript and all the authors edited the manuscript.

WILEY-VCH
